# Complementing Beach Seining With Dive Surveys Improves Fine‐Scale Resolution of 0‐Group Gadoid Distributions in Nearshore Habitats

**DOI:** 10.1002/ece3.71674

**Published:** 2025-07-01

**Authors:** Michelle Lorraine Valliant, Anja Nickel, Ragnar Edvardsson, Guðbjörg Ásta Ólafsdóttir

**Affiliations:** ^1^ Research Centre of the Westfjords University of Iceland Bolungarvik Iceland

**Keywords:** abundance, Atlantic cod, benthic settlement, nursery grounds, recruitment, saithe

## Abstract

Many juvenile fishes use nearshore habitats as critical nursery grounds; however, the distributions of post‐settlement young‐of‐the‐year (0‐group) juvenile Atlantic cod ( *Gadus morhua* ) and saithe ( *Pollachius virens* ) in Icelandic waters remain poorly understood. Catch data from beach seine surveys were combined with fish counts from dive surveys to gain high‐resolution data on the depth distribution (0–21 m) and post‐settlement timing of these species in nearshore habitats. Saithe were most abundant in early summer, and higher numbers were caught in beach seine tows in shallow intertidal algae than observed during dive surveys in deeper (> 5 m) waters. Conversely, Atlantic cod were distributed across the depth range but were generally more abundant in shallower water. Cod became less abundant in dive surveys following an initial peak in July but showed increasing abundance in beach seine catches later in the season. These patterns suggest that settlement occurs later for cod than saithe and that 0‐group cod continue to utilize shallow habitats throughout the study period. Habitat choice, depth‐related predation, or both could explain the contrasting temporal abundance trends for cod observed across the two surveys. Spatial heterogeneity, but no strong habitat associations were found—beyond depth preferences—and no other juvenile gadoid species were caught or observed, confirming that the examined depth range is primarily utilized by 0‐group saithe and cod. Overall, this study contributes to understanding 0‐group gadoid distributions in nearshore nursery habitats by (1) providing detailed depth‐specific abundance estimates previously unavailable in this area, (2) demonstrating key differences in settlement dynamics—including depth and timing—of these species in sympatry, and (3) suggesting depth‐specific predation pressures post‐settlement. This information can support the development of targeted sampling for saithe and cod nursery grounds in Iceland, aiding the assessment of juvenile recruitment into adult populations of these commercially important species.

## Graphical abstract

Located within the Westfjords of Iceland, the fine‐scale depth distribution of juvenile 0‐group gadoids was surveyed in nearshore nursery habitats using beach seine and dive survey methods. The beach seine was used in the intertidal zone, while dive surveys were conducted in the sublittoral zone. The study bridges gaps in understanding gadoid post‐settlement nursery ground use by depth.

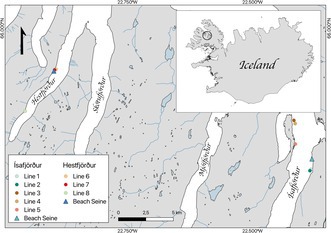

## 1 Introduction

Nursery grounds provide food and shelter from predation for early life stages and are important for supporting recruitment for many commercially fished species (Tupper and Boutilier 1995a, 1995b; Beck et al. 2001; Kamenos et al. 2004). Determining nursery ground area and the timing of habitat use is important to minimize adverse impacts and enhance survival of future harvestable year classes. Following a pelagic larval phase, juvenile gadoids undergo an ontogenetic shift and settle to benthic or benthopelagic habitats (Lomond et al. 1998; Marteinsdóttir et al. 2000; Lough 2010). The timing of young‐of‐the‐year (0‐group) gadoid settlement depends on spawning ground location, spawning time, and oceanographic conditions, and varies both between and within species (Grabowski et al. 2011; Sólmundsson et al. 2015; Endo et al. 2024) often characterized by pulses or gradual change rather than an abrupt shift (Ings et al. 2008; Gregory et al. 2019; Geissinger et al. 2022). The depth of benthic settlement for 0‐group gadoids also varies between species. Haddock ( *Melanogrammus aeglefinus* ) settle at deeper depths, commonly between 50 and 150 m (Bastrikin et al. 2014), while saithe ( *Pollachius virens* ) tend to settle in the shallowest, nearshore habitats within the first few meters of the water column (Clay et al. 1989; Aglen 1995). Atlantic cod ( *Gadus morhua* ) settle at depths ranging from 0 to 200 m (Astthorsson et al. 1994; Berg and Pedersen 2001; Gibb et al. 2007; Dunlop et al. 2022) but settlement depth can also vary between populations and ecotypes (Fevolden et al. 2012; Ólafsdóttir et al. 2023). In shallow waters, small‐scale variations in fish distribution are affected by tides, light cycles, and temperature, resulting in diel migrations to optimize metabolic rates (Clark and Green 1990), foraging, and predator avoidance (Linehan et al. 2001). The diel migration of 0‐group Atlantic cod and saithe is broadly characterized by higher juvenile abundance in intertidal and shallow subtidal waters at night compared to daytime (Keats and Steele 1992; Rangeley and Kramer 1995), leading to changes in depth distribution patterns within a 24‐h period.

Past studies suggest no habitat preference for pre‐settlement larvae and early juveniles, but post‐settlement numbers of 0‐group Atlantic cod tend to be higher in structurally complex habitats, reflecting survival rates or active predator avoidance (Gotceitas and Brown 1993; Gotceitas et al. 1995; Tupper and Boutilier 1995b; Lindholm et al. 1999). Juvenile gadoids inhabit a variety of vegetated and unvegetated habitats (Cote et al. 2013). For example, seagrass beds (Gotceitas et al. 1997; Laurel et al. 2004), cobble substrate (Gotceitas and Brown 1993; Lindholm et al. 1999), kelp (Gotceitas et al. 1995), algae (Borg et al. 1997; Elliott et al. 2016), and maerl (Kamenos et al. 2004). Maerl (rhodolith) beds (unattached *Coralline* spp) are complex habitats consisting of coralline red algal species that form calcareous nodules and can mix with substrates such as cobble, gravel, and coarse sand (Hall‐Spencer 1998; Elliott et al. 2016). Maerl beds in Iceland have not received much attention as nursery grounds but are relatively common in the northwest of the island (Thors 2018; Brynjólfsdóttir 2024) with notable 0‐group gadoid presence (Valliant 2020).

The shallow, structurally complex benthic habitats utilized by juvenile gadoids during their first year of life are often difficult to survey due to rugged terrain and limited accessibility for standard sampling gear, resulting in uncertain estimates of year‐class strength for commercially important species. In Iceland, Atlantic cod and saithe are not well represented in regular fisheries surveys until age three (MFRI 2024a, 2024b). Common methods for monitoring juvenile gadoids include standard trawl‐based surveys, which often fail to capture the smallest 0‐group juveniles due to mesh size limitations and the inaccessibility of shallow nearshore waters to larger vessels. Other gear types, such as gillnets and traps, also face challenges, including high bycatch rates and selectivity biases (e.g., Passarone et al. 2024; Bacheler 2024). As a result, it is difficult to standardize catches across gear types, each being suited to specific habitats and depth ranges. Beach seines are commonly used for 0‐group surveys because their small‐mesh nets are effective in very shallow water and can capture smaller fish. However, they are less effective over structurally complex substrates, are limited to a narrow depth range, and can also result in high bycatch (Cabral et al. 2003). Nevertheless, long‐term beach seine surveys have provided a wealth of information on 0‐group settlement dynamics in shallow waters (< 15 m). For example, the Flødevigen beach seining survey along the Skagerrak coast (Norway) has been conducted since 1919 (Fromentin et al. 2001), and the Fleming survey in Newfoundland (Canada) operated for several multi‐year periods between 1959 and 2002 (Gregory et al. 2004), continuing in localized areas from 1997 to the present (Cote et al. 2013).

Varying timing of juvenile arrival to nursery grounds, post‐settlement survival, and fine‐scale habitat preferences play critical roles in juvenile abundance estimates, necessitating methods that account for temporal and habitat‐specific trends. While technologies such as acoustic telemetry can work well to track these fine‐scale movements for older juveniles (Nickel et al. 2024), tag size limits the applicability for tracking 0‐group individuals (e.g., Brownscombe et al. 2019). Complementary methods such as diving censuses allow for direct, non‐invasive observations in habitats that are otherwise inaccessible to traditional fishing gear, such as rocky substrates or areas with dense or sensitive vegetation. Diving surveys are particularly valuable for assessing microhabitat use and abundance across small‐scale depth gradients and could complement standard surveys. For instance, 0‐group gadoids have been sampled in Icelandic benthic habitats using beach seines and gillnets (Ólafsdóttir et al. 2015, 2023; Nickel 2016) and captured as bycatch in demersal fisheries surveys (Jónsdóttir et al. 2019; Sæmundsson et al. 2020). However, these studies had limitations in estimating 0‐group abundance because of the size selectivity of survey trawls and limited spatial range of beach seines. Importantly, these previous studies have not been able to estimate 0‐group distributions across intermediate depths, ca. 5–30 m. Dive surveys have the potential to provide specific observations across those depths, enabling a more comprehensive understanding of spatial and temporal dynamics of 0‐group gadoids in nearshore nursery grounds.

The objective of this study was to evaluate the depth distributions of 0‐group juvenile gadoids in areas and at depths inaccessible to conventional fisheries trawling and past beach seining surveys. Additionally, the study aimed to compare dive survey observations with multi‐year abundance estimates from beach seining to strengthen inferences about the observed depth distribution and timing of post‐settlement stages. This combined approach can improve estimates of juvenile abundance by accounting for individuals not adequately sampled by standard survey methods.

## 2 Methods

**FIGURE 1:**
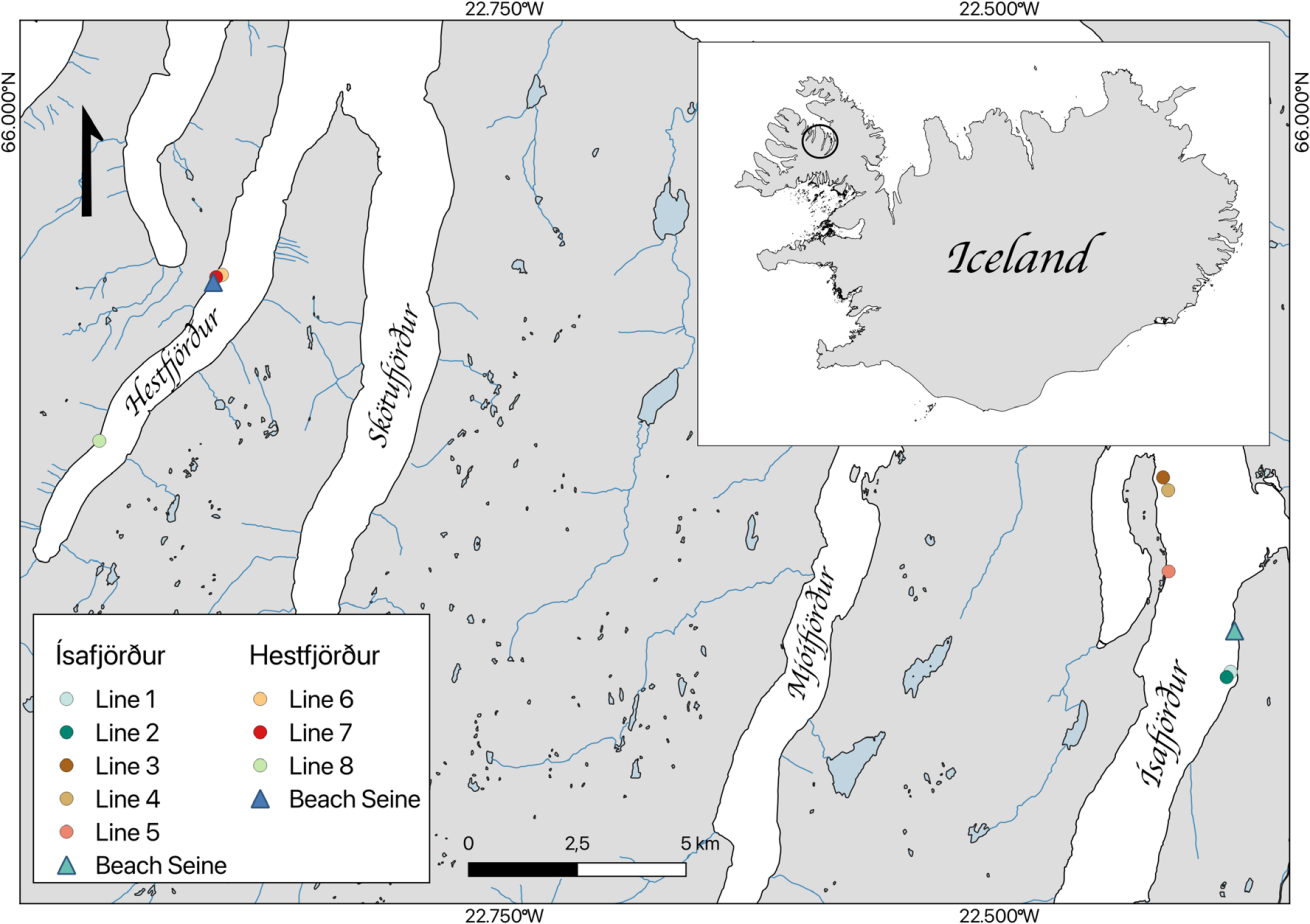
Map overview of study site locations in Hestfjörður and Ísafjörður, Iceland for beach seine and scuba dive surveys.

The study sites were in Ísafjörður and Hestfjörður in northwest Iceland (Figure 1). Glacially carved from the last ice age, these fjords have steep slopes from mountain plateaus to the water's edge. The intertidal habitats feature more gently sloping gravel shores interspersed with rocky patches. While the gravel areas were open habitats with little or no vegetation, the rocky zones were densely covered by rockweed ( *Ascophyllum nodosum* ) and bladderwrack ( *Fucus vesiculosus* ). Moving into the subtidal zone, vegetation was dominated by *Laminaria* spp., badderlocks ( *Alaria esculenta* ), brown filamentous algae, and coralline algae. At depths of 6–10 m, vegetation was sparse, with few kelp species (e.g., *Laminaria* spp.) and coralline algae, but increased variation of substrate compared to the intertidal zone, consisting of coarse sand, silt, gravel, and cobble.

Scuba diving surveys were repeated six times at eight transect lines in the two fjords. The dive surveys were conducted during July–September of 2019 and 2021 (total dive surveys = 48). Each transect line was 50 m long, divided into five 10 m intervals across depths of 6 to 21 m perpendicular to shore (Figure 1, Table 1). The distances between transect lines varied from 100 m to 5 km, and their positions were selected to include a range of representative substrate types. During each dive, the number of fish at each interval (total = 240) was recorded along with the interval depth. As depth was recorded from a dive computer, it represented instantaneous water column depth rather than chartered bathymetry. Sediment classification was based on the Wentworth scale (Wentworth 1922) as coarse sand, cobble, gravel, sand, and silt. If present, maerl was also recorded. Finally, date and timestamp, wind, sea state, tidal level, cloud coverage, underwater visibility, and average temperature were recorded for each dive.

**TABLE 1:** Overview of both dive and beach seining surveys, survey dates, sites, and the number of observations and samples.

	Dates	Site	Observations (*n*)	Line	Cod (*n*)	Saithe (*n*)	Depth (m)	Substrate
Dive survey	09.07.2019–26.09.2019	Ísafjörður (East)	12	Line 1	50	26	7–13	Maerl/Sand/Silt
				Line 2	6	0	7–13	Maerl/Sand/Silt
		Ísafjörður (West)	18	Line 3	5	1	6–17	Maerl/Silt
				Line 4	49	0	6–17	Maerl/Silt
	25.07.2021–30.09.2021			Line 5	21	0	8–18	Coarse Sand/Gravel
		Hestfjörður	18	Line 6	25	8	12–21	Maerl/Gravel/Silt
				Line 7	26	0	11–17	Maerl/Coarse Sand/Silt
				Line 8	21	0	8–17	Coarse Sand/Silt
Beach seine	28.08.2017–26.08.2019	Ísafjörður	4	—	69	165	< 1.5	Algae/Gravel
	13.07.2015–27.09.2019	Hestfjörður	39	—	587	1127	< 1.5	Algae/Gravel

We used juvenile gadoid numbers from beach seine catches (1.5 × 10 m, mesh size = 6 mm) in tidal waters (depth < 1.5 m) to establish a base line of Atlantic cod and saithe numbers in both fjords (Figure 1) following benthic settlement. Beach seining most often took place at low incoming tide; therefore, the measured depth should be interpreted relative to low water level. The beach seining occurred from mid‐July through to late‐September in 2015–2019, coinciding with the time of year of the dive surveys, but only overlapping for the first of the two dive surveys, in 2019 (Table 1). The beach seine sites represented accessible areas with gravel and algal habitats that allowed unrestricted seine tows while still supporting populations of 0‐group gadoids. The beach seining was originally designed to collect enough 0‐group Atlantic cod and saithe for genetic and stable isotope analyses (Nickel 2016; Ólafsdóttir et al. 2023). Up to 10 seine tows were conducted per site to achieve this target. If more than 10 juveniles of both species (the minimum required for analysis) were caught quickly, fewer tows were performed, with a minimum of five tows per site. When catches were dominated by saithe, and enough had been collected, surplus viable saithe were released. The total area towed did not exceed 100 m^2^ per sampling event. Due to this upper limit on the number of juveniles retained, the juvenile counts presented should be interpreted as minimum estimates of abundance for that area, particularly for saithe, which were often abundant but undercounted due to releases.

Data were prepared, analyzed, and visualized using the R environment of Rstudio Desktop application (R Core Team 2023). First, to model the number of juvenile Atlantic cod observed during dive surveys, we fitted a generalized additive mixed model (GAMM) with a zero‐inflated Poisson distribution to account for overdispersion and excess zeros. The response variable was the count of juveniles per transect interval. Day of year (DOY) was included as a linear predictor, and depth was modeled using a thin plate regression spline to capture the non‐linear juvenile distribution across depth. The basis dimension (*k*) was set to 5 to reduce the risk of overfitting across depth. Transect line and year were included as random smooth terms to account for repeated observations on the same transect line and interannual variability in juvenile numbers. Second, to assess the probability of juvenile observation during dive surveys, we fitted generalized linear mixed‐effects models (GLMMs) with a binomial distribution and logit link. The response variable was presence (1) or absence (0) of juveniles in each interval of the transect line. Fixed effects were DOY (scaled) and observed depth per transect line interval. Transect line was included as a random intercept to account for repeated sampling of transect lines and their spatial non‐independence.

Models were fitted separately for each species because of the substantial differences in the number of juvenile observations between species. This approach ensured that the very limited number of saithe observations would not bias parameter estimates or mask species‐specific patterns in a combined analysis. Additionally, although intervals within transects were the unit of observation during the dive surveys, their primary purpose was to assign depth. As a result, transect interval was highly correlated with depth and could not be included as a separate effect in the models. Instead, transect line identity—representing the unit of repeated spatial sampling—was included as a random effect in all models to account for spatial autocorrelation and non‐independence among observations.

For the beach seining data, we fitted a GLMM with a negative binomial distribution to account for overdispersion in the juvenile fish counts. The response variable was the number of juveniles per sampling event, and fixed effects were sampling site (Hestfjörður and Ísafjörður), DOY (scaled), species (Atlantic cod and saithe), and their interaction. To account for variation in cohort size or settlement dynamics among years, we included year as a random intercept. The DOY was scaled prior to analysis to improve model convergence. GAMM fits were done using the package *mgcv* version 1.9‐1 (Wood 2011) and GLMM fits using the package *lme4* version 1.1‐37 (Bates et al. 2015) both in R‐Studio Software (2022, Version 07.1 + 554). Model structure was examined for singular fit and collinearity using base R functions. Model fit was assessed to confirm assumptions of residual normality, spatial and temporal autocorrelation, and appropriate model dispersion using the *DHARMa* package version 0.4.7 (Hartig 2024). Model predictions were visualized using the *marginaleffects* package version 0.24.0 (Arel‐Bundock et al. 2024).

## 3 Results

A total of 239 individual juvenile gadoids, 203 Atlantic cod and 36 saithe, were counted during the dive surveys. All the juveniles were 0‐group individuals (40–100 mm standard length). Juvenile Atlantic cod was observed on 27 out of 48 dives but saithe was only observed on 7 out of 48 dives. There was no correlation between juvenile observation and visibility during the dive (*R* ^2^ = −0.04). The mean number of Atlantic cod observed per dive transect line was 4.00 (SD = 0.44) in Hestfjörður and 4.37 (SD = 3.70) in Ísafjörður, and the mean was skewed by a few large observations. For saithe, these numbers were 0.77 (SD = 0.72) and 0.93 (SD = 0.99) respectively. No other juvenile gadoids were observed during the dives. Other fish species observed were European Plaice ( *Pleuronectes platessa* ) (2 observed), Rock gunnel ( *Pholis gunnellus* ) (6 observed), juvenile flatfish (0‐group, 32 observed), and lastly, a large shoal of 0‐group juvenile herring ( *Clupea harengus* ) (estimated 3000+ individuals) was observed traversing a dive transect.

A total of 1127 saithe and 587 Atlantic cod were beach seined in Hestfjörður in 39 sampling events, and 165 saithe and 69 Atlantic cod were beach seined in Ísafjörður in four sampling events. The mean number of Atlantic cod per sampling event was 17.25 (SD = 17.15) in Ísafjörður and 15.05 (SD = 16.64) in Hestfjörður; mean numbers of caught saithe were 41.25 (SD = 50.76) in Ísafjörður and 28.9 (SD = 24.62) in Hestfjörður. The number of saithe caught with the beach seine was very markedly higher than the number of saithe observed during the dive surveys (Table 1). Catches of other species were rare but included European Plaice ( *Pleuronectes platessa* ), Rock gunnel ( *Pholis gunnellus* ), sculpin ( *Myoxocephalus Scorpius* ), and herring larvae ( *Clupea harengus* ). No other juvenile gadoids were caught with the beach seine.

**FIGURE 2:**
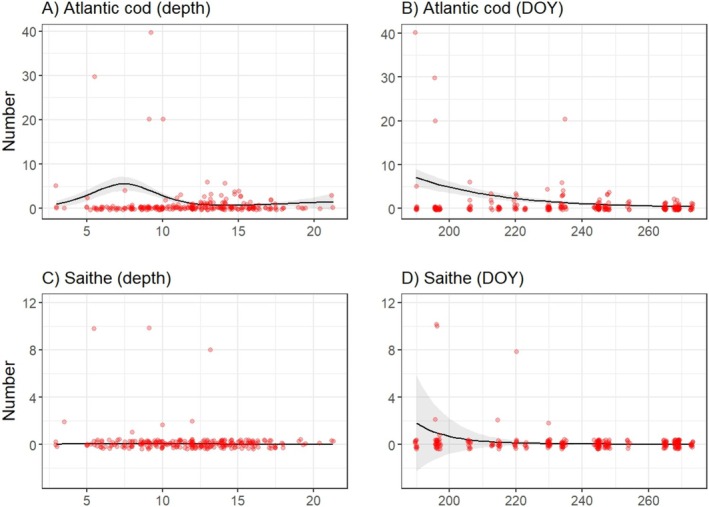
Generalized additive mixed model (GAMM) estimates of the number of 0‐group Atlantic cod (A and B) and saithe (C and D) across depth and day of the year (DOY) based on dive survey counts. Red points represent juvenile observed counts during dives, black lines are model estimates, and the shaded areas show the error around the estimate. DOY *x*‐axis tick marks represent July 19, August 8, August 28, and September 17.

**FIGURE 3:**
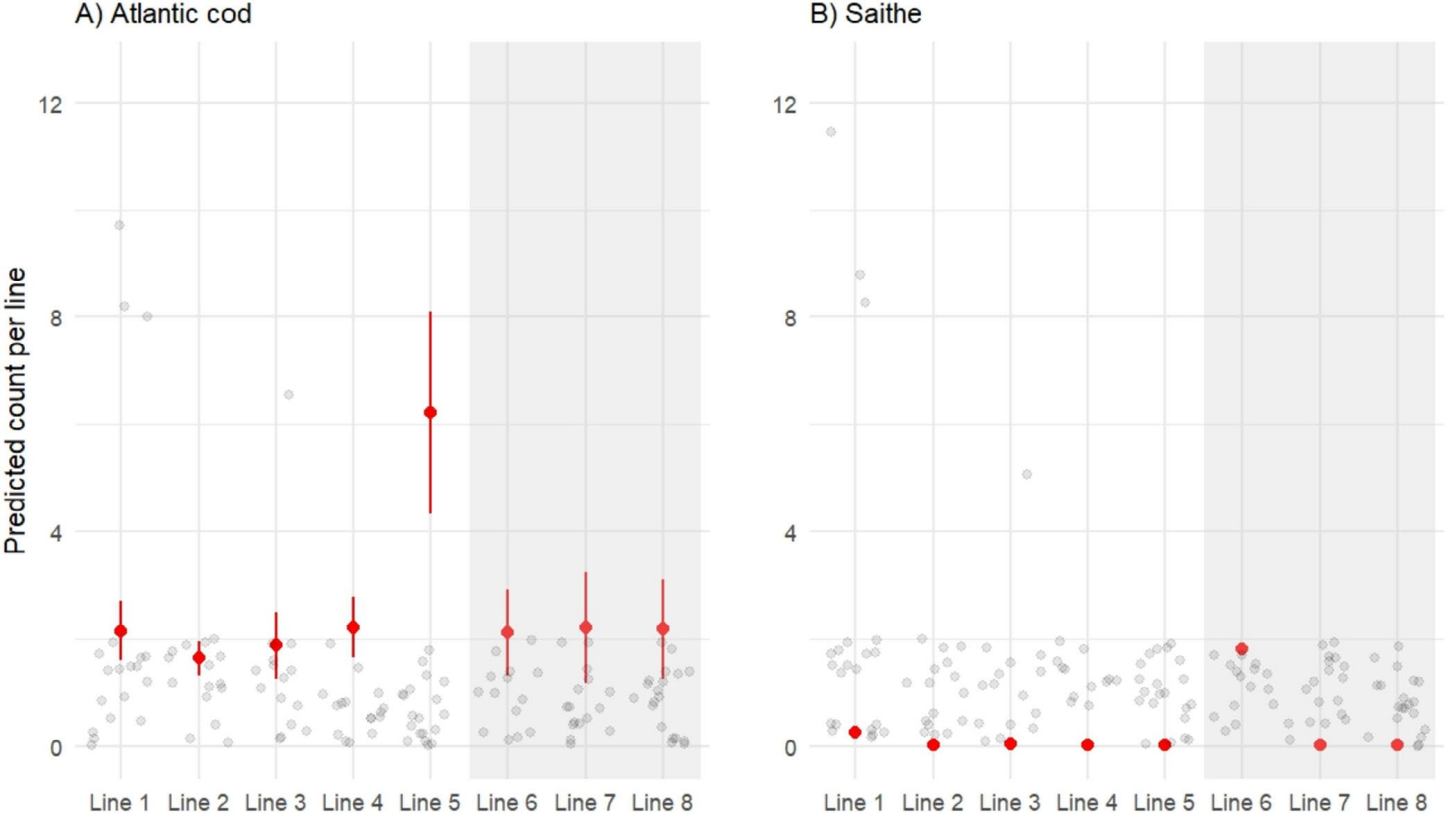
Generalized additive mixed model (GAMM) estimates of the number of 0‐group Atlantic cod (A) and saithe (B) counted during dives on each transect line. To facilitate comparison with beach seine numbers caught the figure presents counts per line not per intercept as that area of observation is more comparable to the areas sampled with the beach seine. Gray points represent juvenile counts during dives, red points are model estimates shown with standard error. The shaded area highlights the different survey years as lines 6–8 were surveyed in 2021.

GAMMs modeling juvenile abundance from dive survey data showed significant declines in abundance for both species following benthic settlement (Table 2, Figure 2). For cod, depth had a non‐linear effect on abundance (Table 2, Figure 2). With peak densities at depths between 4 and 10 m. In contrast, depth was not a significant predictor of saithe abundance (Table 2, Figure 2). Significant variation among transects was found for both species (Table 2), suggesting spatial heterogeneity in juvenile densities. No significant interannual variation (between the two survey years) was detected for either species. GLMMs assessing the probability of juvenile presence revealed no significant effects of depth or DOY for either Atlantic cod or saithe (Figure 3). However, in both models, there was substantial variation among transect lines (random intercept variance = 0.933), again suggesting spatial heterogeneity in juvenile distribution.

**TABLE 2:** Estimates from the generalized additive mixed model (GAMM) examining 0‐group juvenile numbers counted during the dive surveys.

	Parametric coefficients	Estimate	SE	*t*	*p*	Smooth term	edf	Ref. df	*F*	*p*
(A) Atlantic cod	Intercept	9.654	0.612	15.784	**0.000**					
	DOY	−0.037	0.003	−14.103	**0.000**					
						s(depth)	3.843	3.985	120.555	**0.000**
						s(line)	5.910	7.000	145.088	**0.000**
						s(year)	0.000	1.000	0.000	0.815
(B) Saithe	Intercept	11.390	4.241	2.686	**0.007**					
	DOY	−0.065	0.018	−3.636	**0.000**					
						s(depth)	2.475	2.953	6.939	0.108
						s(line)	4.073	7.000	14.524	**0.001**
						s(year)	0.000	1.000	0.000	0.518

*Note:* (A) Atlantic cod deviance explained 0.651; (B) Saithe deviance explained 0.824. Significant values are boldfaced.

**FIGURE 4:**
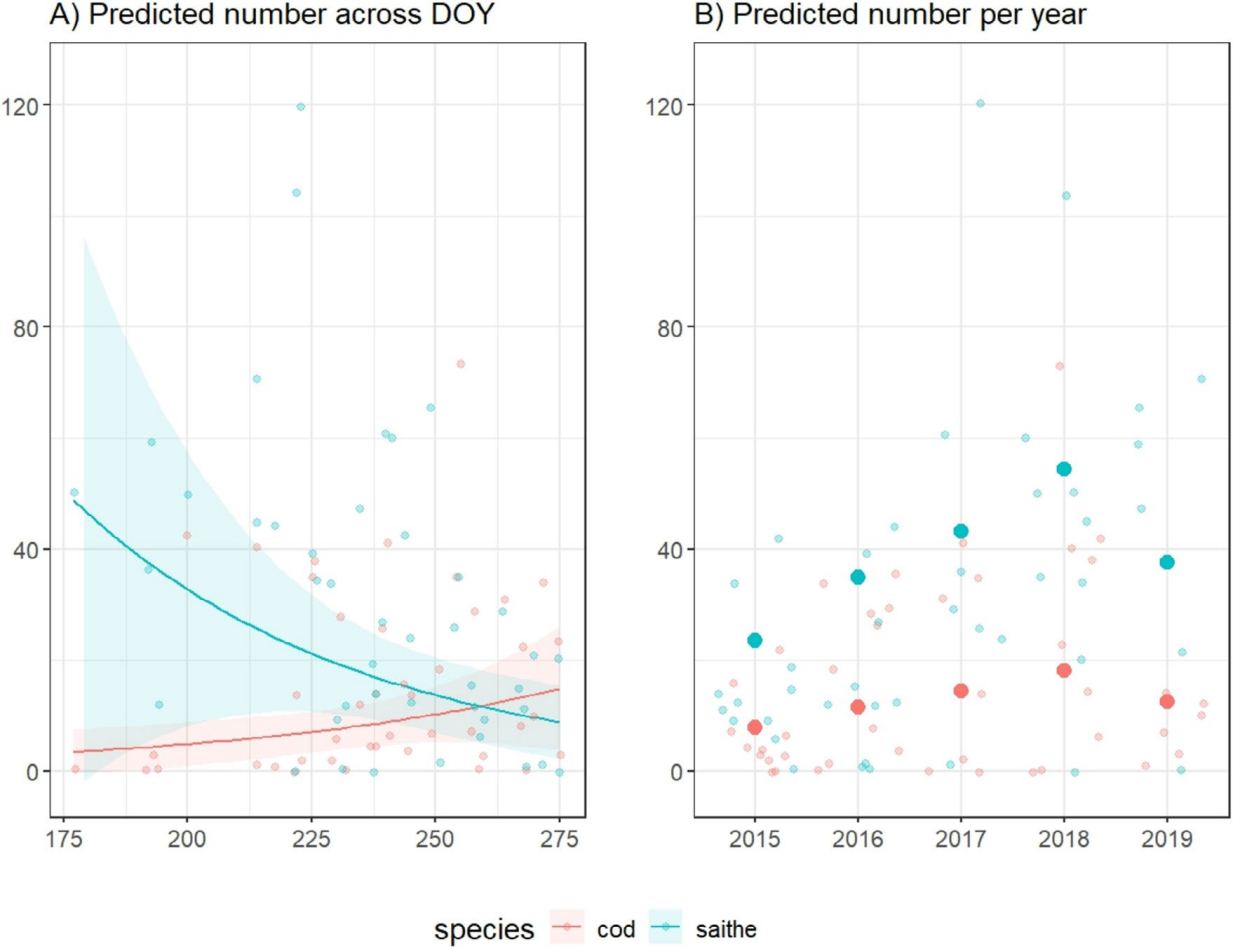
Observed and estimated number of 0‐group Atlantic cod and saithe caught per sampling event of the beach seining survey shown for (A) day of the year (DOY) and (B) across years. The observed data are presented as lighter colored points and the predictions are shown as lines (A) or larger, darker points (B) with the associated errors represented by shaded areas. DOY *x*‐axis tick marks represent June 24, July 19, August 13, September 7, and October 2.

For the beach seining data, the GLMM revealed significant effects of species and its interaction with DOY on juvenile abundance. Specifically, saithe had higher overall juvenile counts compared to Atlantic cod (Table 3, Figure 4), but the negative interaction between DOY and species (Table 3, Figure 4) indicates that the abundance of cod, but not saithe, increased across the sampling period. There was no significant difference between the two sampling sites. The random effect of year (variance = 0.349) suggested some interannual variability in juvenile counts.

**TABLE 3:** Estimates from the generalized linear mixed model (GLMM) on the numbers of Atlantic cod and saithe caught with a beach seine.

Fixed effects	Estimate	SE	*z*	*p*	Random effect	Variance	SD
Intercept	2.672	0.450	5.934	**0.000**			
Site: Hestfjörður	−0.057	0.426	−0.134	0.894			
DOY	0.359	0.205	1.754	0.080			
Species: saithe	0.670	0.242	2.767	**0.006**			
DOY: species	−0.780	0.276	−2.823	**0.005**			
					Year	0.122	0.350

*Note:* Significant values are boldfaced.

## 4 Discussion

We found that the 0‐group saithe associated almost exclusively with the shallow intertidal algae and were rarely seen in deeper waters. Conversely, 0‐group Atlantic cod were distributed across the depths examined but most common in shallower waters (< 10 m). Specifically, we observed that Atlantic cod became more frequent in the shallow algae beds moving from July to September, marking the timing of benthic settlement, while saithe had arrived earlier at the nursery grounds. No other gadoid juveniles were caught or observed during the study suggesting that during this time of year the examined depth range (0–21 m) is used exclusively by 0‐group saithe and Atlantic cod. Importantly, the current study provides novel insights into spatiotemporal distribution of 0‐group gadoids by extending estimates to depths that were out of range for previous sampling efforts in this area (Ólafsdóttir et al. 2023) and supports depth‐specific associations by active habitat choice or predation (Tupper and Boutilier 1995a).

Juvenile numbers following benthic settlement reflect the timing of spawning as well as variation in physical oceanography and retention post‐settlement (Lough et al. 1989; Morgan et al. 2013) and for Atlantic cod, immigration to nursery grounds can occur in pulses or over a prolonged period (Geissinger et al. 2022). In the current study, many more juveniles were observed with the dive surveys in July compared to August and September. This largely reflected sightings of larger juvenile aggregations in July, followed by more frequent individual sightings or smaller aggregations in late August and September. For Atlantic cod, this pattern may reflect peak arrival time of pre‐settlement juveniles and coincide with behavioral changes from pelagic shoaling to establishing territories (Tupper and Boutilier 1995b; Lomond et al. 1998; Anderson et al. 2007) and more solitary behavior (Lomond et al. 1998; Laurel et al. 2003). Conversely, the beach seine numbers of 0‐group Atlantic cod were higher in September than in July, most likely reflecting the peak time of settlement into benthic and demersal habitats, suggesting that 0‐group Atlantic cod were more likely to aggregate at intertidal habitats and/or their survival in these habitats was higher than at more depth during this time in their life history.

Saithe spawn earlier than Atlantic cod (Armannsson et al. 2007; Marteinsdóttir and Björnsson 1999), and 0‐group saithe were present at demersal nursery grounds earlier in the season. The number of 0‐group saithe caught in beach seine surveys declined from July to September, and results from the dive surveys did not indicate a shift to deeper waters during this period. Saithe are classified as a semi‐pelagic species not bound to demersal habitats, and the current results support the interpretation that their movement away from intertidal nurseries does not follow a vertical migration pattern. It is possible that the disappearance of saithe reflects thermoregulation, achieved not through vertical migration but by leaving shallow demersal nursery grounds for cooler water masses. Previous work has shown that 1‐year‐old saithe in summer foraging grounds were more sensitive to high summer temperatures than Atlantic cod of the same age, leading to their earlier departure from in‐fjord nurseries (Nickel et al. 2024). However, it is also possible that the observed decline in 0‐group saithe reflects high predation pressure, as suggested for juvenile gadoids by Bergstad et al. (1987), although this would require substantially higher predation rates on saithe than on cod. In either case, the combined survey methods used in the present study support the conclusion that 0‐group saithe were highly associated with intertidal algal habitats at shallower depths and were more abundant earlier in the summer compared to Atlantic cod.

In contrast to the shoaling preference of saithe, juvenile Atlantic cod tend to be more solitary and show active habitat selection to avoid predators (Partridge et al. 1980). Previous studies estimating juvenile gadoid densities post‐settlement have suggested that densities depend on the complexity of habitat (Fromentin et al. 2001; Warren et al. 2010; Dunlop et al. 2022). Tupper and Boutilier (1995a) found that post‐settlement survival depended on the rugosity and complexity of the habitat, and 0‐group juvenile Atlantic cod survival was lowest in sandy substrate and highest for reefs (Tupper and Boutilier 1995a). Similarly, Laurel et al. (2003) found that predation risk for 0‐group cod was higher in small eelgrass patches and over unvegetated sand than in larger eelgrass patches—highlighting the role of habitat structure and predator distribution in shaping juvenile cod survival. In the present study, habitat‐specific predation could explain the contrasting temporal patterns between the dive and beach seining surveys, with a temporal decline in cod numbers during dive surveys coinciding with the time of increase in beach seine catches. It is likely that aggregating in or near intertidal algae provides juvenile cod with shelter from predation.

Despite spatial heterogeneity in juvenile numbers, we did not observe habitat associations—beyond depth preferences—as the variation in abundance between transects appears sporadic and largely reflects the early occurrences of larger shoals. However, the relatively uniform substrate across transect lines and the limited number of dive observations limit strong conclusions on habitat. The habitats surveyed were not highly vegetated, but maerl was often present. Maerl beds, as well as patchy and fragmented maerl (e.g., encrusting coralline algae), are common in NW‐Iceland (Thors 2018; Brynjólfsdóttir 2024). In the dive surveys, several larger juvenile aggregations were observed sheltering among the maerl thalli early in the season, suggesting that habitat diversity and structural rugosity may be important for the habitat transitions of 0‐group Atlantic cod. Although those observations did not allow estimation of the role of maerl as nursery grounds, they warrant further examination of this association in Icelandic waters, especially coupled with previous dive surveys showing significantly more juvenile gadoids over maerl than rock/gravel (Kamenos et al. 2004).

It is difficult to compare fish abundance across different surveys, and any comparison of the number of juveniles across the dive and beach seining surveys presented here should consider confounding factors, including gear selectivity, diel migration, interannual variation in cohort size, and visibility during dive surveys. First, while the fine mesh beach seine likely sampled all 0‐group juveniles encountered by the net, dive survey counts may depend on visibility and fish behavior. Scuba dive surveys in Iceland are commonly affected by low visibility (Edvardsson and Egilsson 2015) and although visibility did not significantly explain juvenile observation numbers in the current study, it was generally low (between 2 and 5 m), and this could result in fewer observations as the fish hide or swim out of view in response to the scuba diver's presence (Dickens et al. 2011; La Manna et al. 2021). Observations during the present study confirmed that juvenile gadoids often swam closely along the bottom substrate and paused next to it until the diver approached (Valliant, personal observation), behavior reflecting anti‐predator strategies previously described for Atlantic cod (Lindholm et al. 1999; Gotceitas and Brown 1993). Such behavior, combined with low visibility, could contribute to lower observed counts in dive surveys, as fish either concealed themselves within the substrate (Gregory and Anderson 1997) or moved away from the diver's field of view. However, this would more likely affect Atlantic cod estimates than saithe estimates due to innate behavioral differences between the two species; saithe are more likely to shoal in response to predators. Diel migration can also skew abundance estimates of fish in the nearshore habitats (Gaelzer et al. 2006; Ley and Halliday 2007). The diel migration of 0‐group Atlantic cod and saithe can be broadly characterized by higher juvenile abundance in shallow waters at night compared to daytime (Keats and Steele 1992; Rangeley and Kramer 1995). However, all surveys in the present study were conducted during daylight hours, typically around midday, also with minimal variation in tidal range.

Finally, high interannual variation in year‐class strength is documented for both Atlantic cod and saithe (e.g., Campana et al. 1989; Campana 1996; Kristiansen et al. 2011; Nedreaas 1995) and since there was not full annual overlap between the beach seine and the dive surveys, this could potentially affect any comparison of juvenile numbers. Nevertheless, given the relatively consistent estimates across the 5 years of beach seining, this is unlikely to alter the conclusions of the present study. The dive estimates for saithe were close to zero for all transect lines and both years (over an approximate surveyed area of 100 m^2^) but in no year lower than 15 individuals per 100 m^2^ in the beach seine surveys. For Atlantic cod, the estimated number (averaged within year) was lowest at 10 juveniles per beach seining event (ca. 100 m^2^) whereas the estimates from the dive surveys were 2–6 juveniles per 100 m^2^ (averaged across depth and DOY). Therefore, having considered these confounding factors, we concluded that it is unlikely that they explain the high difference in the numbers of saithe observed in beach seining and dive surveys. Our conclusions on Atlantic cod dynamics were only based on interpreting temporal and depth‐related variations within each survey method, rather than on comparisons of numbers across survey types.

To conclude, the present study documents interspecific variation in the temporal and spatial use of nearshore nurseries and highlights the strong reliance of 0‐group saithe on intertidal and subtidal zones during their early demersal life stage. Understanding where productive capacity of nursery grounds lies across nearshore habitats is critical, as effective sampling of juvenile life stages may provide predictive power to estimate adult population abundance (e.g., Lunzmann‐Cooke et al. 2021; Laurel et al. 2017). The current results suggest that targeted beach seining surveys could provide reliable estimates of 0‐group saithe and coastal cod abundance during their initial arrival at nursery grounds. However, we recommend incorporating genetic sampling across depth strata in future fieldwork to resolve ecotype‐specific spatial and temporal variance. Previous research has shown that more than 90% of beach seined 0‐group Atlantic cod in these fjords are genetically coastal ecotype and that the Atlantic cod offshore ecotype was much more abundant in bottom trawl surveys at depths of more than 30 m (Ólafsdóttir et al. 2023). The present study fills a key observational gap by providing numerical estimates of 0‐group gadoids across intermediate depths, but more comprehensive sampling and genotyping are needed to fully resolve the spatial and temporal dynamics of post‐settlement 0‐group Atlantic cod in relation to ecotype.

## Author Contributions

**Michelle Lorraine Valliant:** conceptualization (equal), data curation (equal), formal analysis (equal), investigation (equal), methodology (equal), resources (equal), software (equal), validation (equal), visualization (equal), writing – original draft (equal), writing – review and editing (equal). **Anja Nickel:** conceptualization (equal), data curation (equal), formal analysis (equal), investigation (equal), methodology (equal), resources (equal), software (equal), validation (equal), visualization (equal), writing – original draft (equal), writing – review and editing (equal). **Ragnar Edvardsson:** conceptualization (equal), data curation (equal), formal analysis (equal), funding acquisition (equal), investigation (equal), methodology (equal), project administration (equal), resources (equal), software (equal), supervision (equal), validation (equal), visualization (equal), writing – original draft (equal), writing – review and editing (equal). **Guðbjörg Ásta Ólafsdóttir:** conceptualization (equal), data curation (equal), formal analysis (equal), funding acquisition (lead), investigation (equal), methodology (equal), project administration (lead), resources (lead), software (equal), supervision (lead), validation (equal), visualization (equal), writing – original draft (equal), writing – review and editing (equal).

## Ethics Statement

The beach seining was licensed by the Ministry of Fisheries. To minimize stress and suffering, fish were quickly collected from the seine, moved into containers filled with fresh sea water, and subsequently anesthetized by overexposure to phenoxyethanol. Ecological sampling with humane lethal endpoints is not subject to animal welfare permits by Icelandic law (Act 55/2013). The animals in the dive surveys were observed in their natural habitat without significant disturbance from the scuba dive observations. Therefore, no animal welfare laws, policies, or permits were applicable.

## Conflicts of Interest

The authors declare no conflicts of interest.

## Supporting information


**Table S1.** Data file used for analysis of Atlantic cod and saithe occurrence and observation depth use.

**Table S2.** Data file used for analysis of beach seine count of Atlantic cod and saithe.

## Data Availability

All data used in this publication is available in the Tables S1 and S2.
